# GLP-2 Improves Hepatic Inflammation and Fibrosis in *Mdr2*^*-/-*^ Mice Via Activation of NR4a1/Nur77 in Hepatic Stellate Cells and Intestinal FXR Signaling

**DOI:** 10.1016/j.jcmgh.2023.08.003

**Published:** 2023-08-10

**Authors:** Claudia D. Fuchs, Thierry Claudel, Veronika Mlitz, Alessandra Riva, Moritz Menz, Ksenia Brusilovskaya, Felix Haller, Maximilian Baumgartner, Philipp Königshofer, Lukas W. Unger, Wilhelm Sjöland, Hubert Scharnagl, Tatjana Stojakovic, Georg Busslinger, Thomas Reiberger, Hanns-Ulrich Marschall, Michael Trauner

**Affiliations:** 1 Hans Popper Laboratory of Molecular Hepatology, Division of Gastroenterology and Hepatology, Department of Internal Medicine III, Medical University of Vienna, Vienna, Austria; 2 Division of Gastroenterology and Hepatology, Department of Internal Medicine III, Medical University of Vienna, Vienna, Austria; 3 CeMM Research Center for Molecular Medicine of the Austrian Academy of Sciences, Vienna, Austria; 4 Vienna Experimental Hepatic Hemodynamic Lab (HEPEX), Medical University of Vienna, Vienna, Austria; 5 Christian Doppler Laboratory for Portal Hypertension and Liver fibrosis, Medical University of Vienna, Vienna, Austria; 6 Division of Visceral Surgery, Department of General Surgery, Medical University of Vienna, Vienna, Austria; 7 Department of Molecular and Clinical Medicine/Wallenberg Laboratory, Sahlgrenska Academy, University of Gothenburg, Gothenburg, Sweden; 8 Clinical Institute of Medical and Chemical Laboratory Diagnostics, Medical University of Graz, Graz, Austria; 9 Clinical Institute of Medical and Chemical Laboratory Diagnostics, University Hospital Graz, Graz, Austria

**Keywords:** Fibrosis, Bile Acid Homeostasis, Nuclear Binding, FGF15/19

## Abstract

**Background & Aims:**

Glucagon-like peptide (GLP)-2 may exert antifibrotic effects on hepatic stellate cells (HSCs). Thus, we aimed to test whether application of the GLP-2 analogue teduglutide has hepatoprotective and antifibrotic effects in the *Mdr2/Abcb4*^*-/-*^ mouse model of sclerosing cholangitis displaying hepatic inflammation and fibrosis.

**Methods:**

*Mdr2*^*-/-*^ mice were injected daily for 4 weeks with teduglutide followed by gene expression profiling (bulk liver; isolated HSCs) and immunohistochemistry. Activated HSCs (LX2 cells) and immortalized human hepatocytes and human intestinal organoids were treated with GLP-2. mRNA profiling by reverse transcription polymerase chain reaction and electrophoretic mobility shift assay using cytosolic and nuclear protein extracts was performed.

**Results:**

Hepatic inflammation, fibrosis, and reactive cholangiocyte phenotype were improved in GLP-2-treated *Mdr2*^*-/-*^ mice. Primary HSCs isolated from *Mdr2*^*-/-*^ mice and LX2 cells exposed to GLP-2 in vitro displayed significantly increased mRNA expression levels of NR4a1/Nur77 (*P* < .05). Electrophoretic mobility shift assay revealed an increased nuclear NR4a1 binding after GLP-2 treatment in LX2 cells. Moreover, GLP-2 alleviated the Tgfβ-mediated reduction of NR4a1 nuclear binding activity. In vivo, GLP-2 treatment of *Mdr2*^*-/-*^ mice resulted in increased intrahepatic levels of muricholic acids (accordingly Cyp2c70 mRNA expression was significantly increased), and in reduced mRNA levels of Cyp7a1 and FXR. Serum Fgf15 levels were increased in *Mdr2*^*-/-*^ mice treated with GLP-2. Accordingly, GLP-2 treatment of human intestinal organoids activated their FXR-FGF19 signaling axis.

**Conclusions:**

GLP-2 treatment increased NR4a1/Nur77 activation in HSCs, subsequently attenuating their activation. GLP-2 promoted intestinal Fxr-Fgf15/19 signaling resulting in reduced Cyp7a1 and increased Cyp2c70 expression in the liver, contributing to hepatoprotective and antifibrotic effects of GLP-2 in the *Mdr2*^*-/-*^ mouse model.

## Graphical abstract

**Figure:**
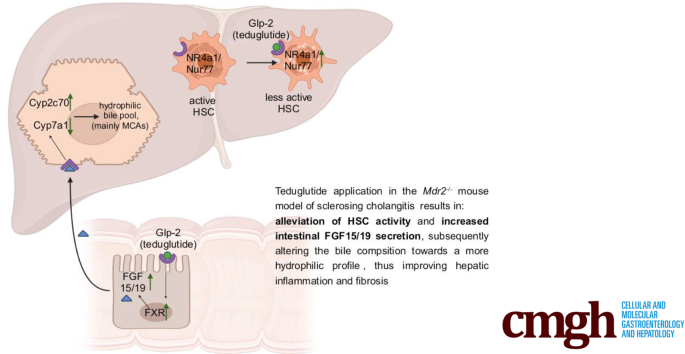

**Summary**

In the *Mdr2*^*-/-*^ mouse model of sclerosing cholangitis GLP-2 was identified to activate NR4a1 in hepatic stellate cells, thus ameliorating their activation. Furthermore, GLP-2 increased intestinal FXR signaling. Together these features result in improved hepatic inflammation and fibrosis.

As a result of impaired biliary phospholipid secretion and subsequent increase of free nonmicellar bound (potentially toxic) biliary bile acid (BA) concentration, the *Mdr2/Abcb4*^*-/-*^ mouse model of sclerosing cholangitis develops pericholangitis, ductular proliferation, reactive cholangiocyte phenotype, and onion skin–type periductal fibrosis,^1^ reflecting central morphologic features of primary sclerosing cholangitis (PSC).^2^^,^^3^ Activation of hepatic stellate cells (HSC) has been shown to be the primary source to promote chronic cholestatic liver fibrosis.^4^^,^^5^ Therefore, HSCs may be a potential pharmacologic target for new therapy strategies to combat cholangiopathies, such as PSC. Recently, mRNA sequencing identified the presence of the glucagon-like peptide (GLP)-2 receptor in HSC,^6^^,^^7^ and possibly hepatocytes.^8^ Moreover, it has been demonstrated that loss of GLP-2 receptor signaling in HSCs led to their activation in steatohepatitis,^6^ indicating a novel hepatic function of GLP-2 beyond its established role in the intestine.^9^ Furthermore, nuclear receptor subfamily 4 group a member 1 (NR4a1)/Nur77 has been identified as key regulator of hepatic fibrosis and its suppression is heavily involved in the activation process of HSCs.^10^ Activated HSCs have been also shown to secrete proinflammatory chemokines and cytokines^11^ inducing hepatic inflammation^11^ and reactive cholangiocyte phenotype.^12^ Therefore, the GLP-2 analogue teduglutide, known for its proproliferative effect in the gut, thus reducing the requirement for parenteral nutrition in patients with short-bowel syndrome,^13^ could be an interesting pharmacologic approach to counteract liver injury. In the present study we investigated whether treatment with the GLP-2 analogue teduglutide improves liver injury in the *Mdr2/Abcb4*^*-/-*^ mouse model of sclerosing cholangitis possibly via activation of NR4a1/Nur77 in HSCs.

## Results

### GLP-2 Treatment Improves Hepatic Inflammation and Fibrosis in *Mdr2*^*-/-*^ Mice

**Figure 1:**
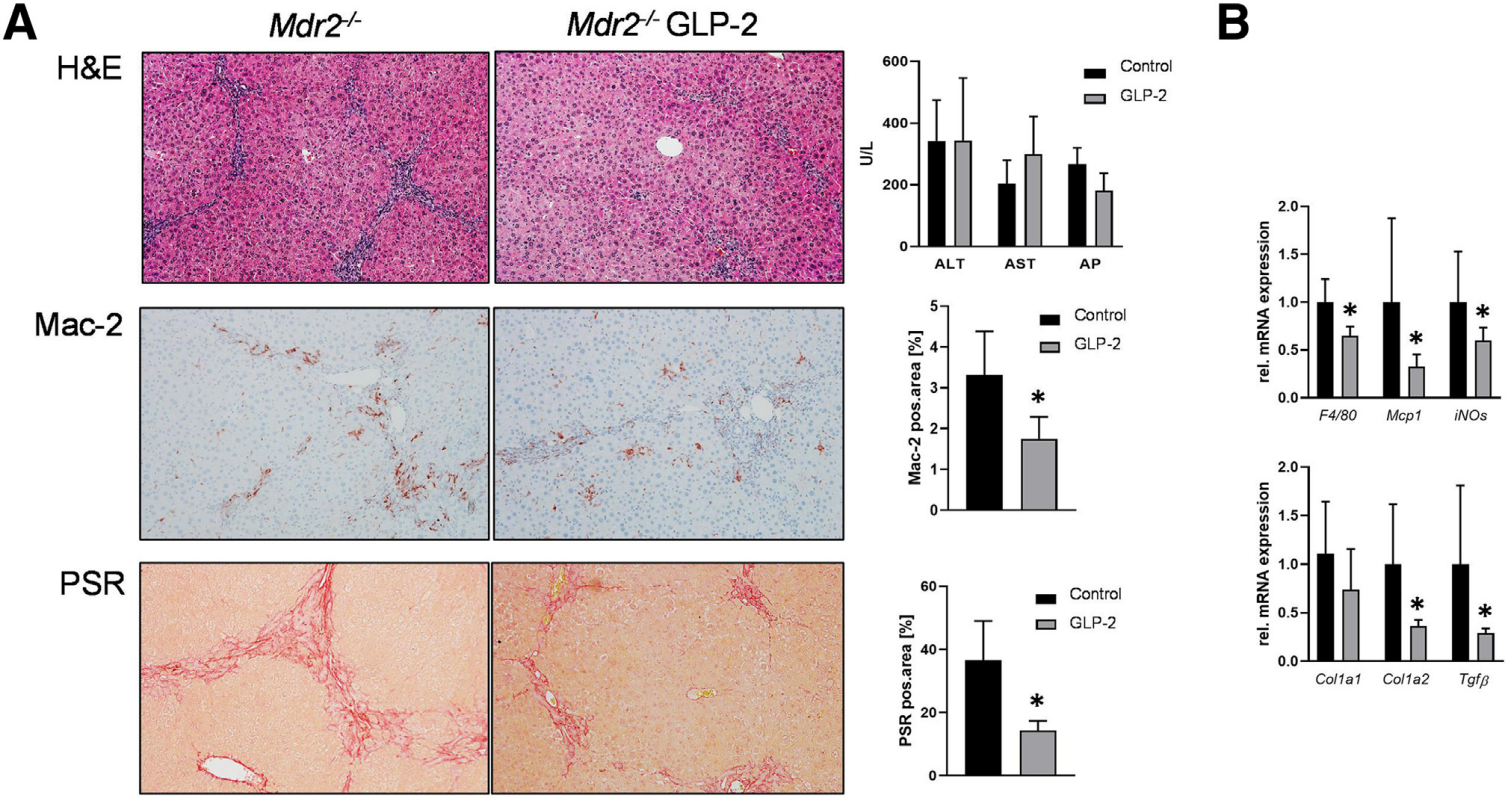
**GLP-2 treatment improves hepatic inflammation and fibrosis in *Mdr2***^***-/-***^**mice.** (*A*) Representative hematoxylin-eosin images (×10 magnification) with markedly improved liver histology in *Mdr2*^*-/-*^ mice treated with GLP-2. Serum biochemistry reflects unchanged levels of transaminases (alanine aminotransferase, aspartate aminotransferase) and alkaline phosphatase. Representative Mac-2 and Sirius red (SR) pictures (×10 magnification) show reduced hepatic inflammation and fibrosis in *Mdr2*^*-/-*^ mice treated with GLP-2. (*B*) Real-time polymerase chain reaction was used to assess the mRNA expression of inflammatory and fibrotic markers *F4/80*, *Mcp1*, *iNOs*, *Col1a1*, *Col1a2*, and *Tgfβ*, which were significantly reduced in *Mdr2*^*-/-*^ mice treated with GLP-2. mRNA expression values were normalized against *36b4* levels and are shown relative to the expression level in *Mdr2*^*-/-*^ control subjects. ∗Significant difference from *Mdr2*^*-/-*^ control mice; *P* < .05. Computational analysis of histologic pictures was done via Image J 1.51j8. ALT, alanine aminotransferase; AP, alkaline phosphatase; AST, aspartate aminotransferase.

To investigate whether the GLP-2 analogue teduglutide has a positive effect on the liver phenotype of *Mdr2*^*-/-*^ mice, GLP-2 was administered via intraperitoneal injection for a time period of 4 weeks. Although serum levels of liver transaminases alanine aminotransferase and aspartate aminotransferase and alkaline phosphatase remained unchanged between untreated control and GLP-2-injected *Mdr2*^*-/-*^ mice, liver phenotype of *Mdr2*^*-/-*^ mice (eg, pericholangitis) improved in presence of GLP-2 (Figure 1*A*). Accordingly, hepatic inflammation and hepatic fibrosis were ameliorated in GLP-2-treated animals, reflected by significantly lower numbers of MAC-2-positive cells (MAC-2 immunohistochemistry) and markedly lower amount of collagen (Sirius red staining) in the livers of these mice (Figure 1*A*). In line with these findings, gene expression profiling revealed a clear reduction of mRNA levels of inflammatory markers *F4/80*, *Mcp1*, and *iNOs* and of the fibrotic markers *Col1a1*, *Col1a2*, and *Tgfβ* (Figure 1*B*).

### GLP-2 Treatment Improves Reactive Cholangiocyte Phenotype in *Mdr2*^*-/-*^ Mice

**Figure 2:**
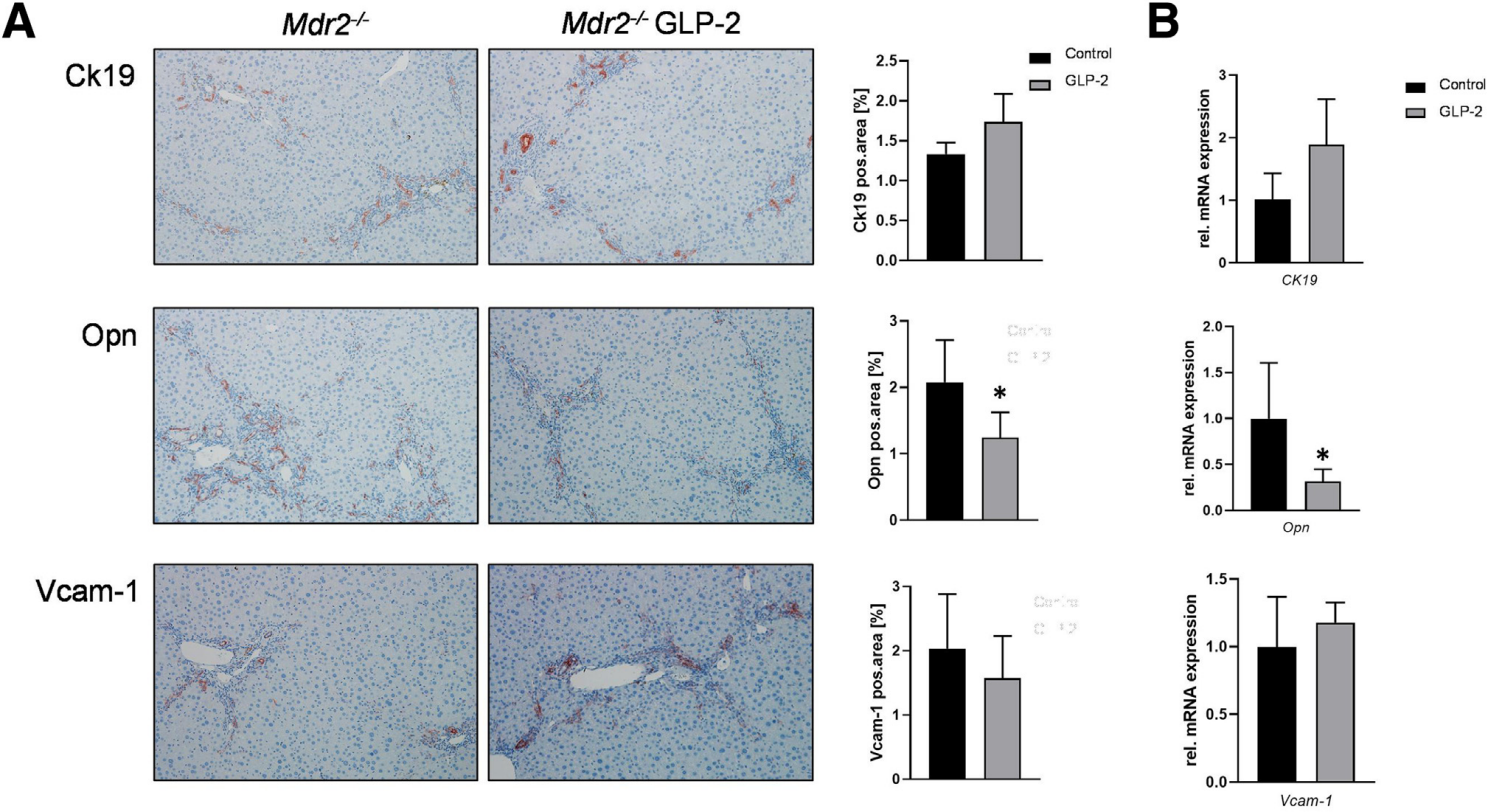
**GLP-2 treatment improves reactive cholangiocyte phenotype in *Mdr2***^***-/-***^**mice.** (*A*) Representative Ck19 images (×10 magnification) show tendentially increased cholangiocyte proliferation in GLP-2-treated *Mdr2*^*-/-*^ animals, whereas representative osteopontin (Opn) images (×10 magnification) reflect reduced Opn secretion from cholangiocytes. Vcam-1 images (×10 magnification) remained unchanged among the groups. (*B*) Real-time polymerase chain reaction was used to assess the mRNA expression of *Ck19*, *Opn*, and *Vcam-1*. Whereas *Ck19* expression tended to be increased, *Opn* levels were reduced and *Vcam-1* remained unchanged in *Mdr2*^*-/-*^ mice treated with GLP-2. mRNA expression values were normalized against *36b4* levels and are shown relative to the expression level in *Mdr2*^*-/-*^ control animals. ∗Significant difference from *Mdr2*^*-/-*^ control mice; *P* < .05. Computational analysis of histologic pictures was done via Image J 1.51j8.

Because the development of hepatic inflammation and fibrosis under cholestatic conditions is linked to a reactive cholangiocyte phenotype,^14^ respective markers were investigated by immunohistochemistry (Figure 2*A*) and real-time polymerase chain reaction (Figure 2*B*). Although Ck19 (marker for cholangiocyte proliferation) staining and mRNA levels tended to be increased, osteopontin was significantly decreased in *Mdr2*^*-/-*^ mice treated with GLP-2. Of note, Vcam-1 remained unchanged at mRNA and at protein level (Figure 2*A* and *B*). Together these data indicate that despite a trend for increased ductular proliferation, treatment with the GLP-2 analogue teduglutide reduced secretion of proinflammatory markers, such as osteopontin and Vcam-1, from cholangiocytes in the *Mdr2*^*-/-*^ mouse model of sclerosing cholangitis.

### GLP-2 Treatment Increases the Transcription of NR4a1/Nur77 in Primary Hepatic Stellate Cells

**Figure 3:**
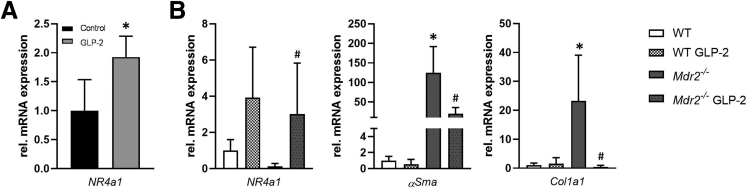
**NR4a1/Nur77 mRNA expression is significantly increased in primary hepatic stellate cells because of GLP-2 treatment.** (*A*) Real-time polymerase chain reaction was used to assess the mRNA expression of *NR4a1/Nur77* in whole liver homogenate. mRNA expression values were normalized against *36b4* levels and are shown relative to expression level in *Mdr2*^*-/-*^ control subjects. ∗Significant difference from *Mdr2*^*-/-*^ control mice; *P* < .05 (*B*) Real-time polymerase chain reaction was used to assess the mRNA expression of *NR4a1/Nur77 αSma* and *Col1a1* in primary hepatic stellate cells isolated from wild-type (WT) and *Mdr2*^*-/-*^ mice with and without GLP-2 treatment. GLP-2 treatment significantly increased expression levels of *Nr4a1/Nur77*. *αSma* and *Col1a1* mRNA levels were significantly reduced in primary hepatic stellate cells isolated from *Mdr2*^*-/-*^ mice treated with GLP-2 compared with untreated *Mdr2*^*-/-*^ mice. mRNA expression values were normalized against *36b4* levels and are shown relative to the expression level in WT control animals. ^#^Significant difference from *Mdr2*^*-/-*^ control mice; *P* < .05.

Because GLP-2 activates NR4a1/Nur77 in HSCs, thereby counteracting their activation,^15^ we subsequently investigated *NR4a1* mRNA expression in whole liver lysates (Figure 3*A*) and in primary HSCs isolated from wild-type and *Mdr2*^*-/-*^ mice subjected to GLP-2 treatment (Figure 3*B*). *NR4a1* transcription was increased in whole liver lysates and in isolated HSCs from GLP-2-treated animals. In line with this, α*Sma* and *Col1a1* expression was reduced in HSCs in *Mdr2*^*-/-*^ mice subjected to GLP-2 treatment (Figure 3*B*).

### GLP-2 Treatment Restores Tgfβ-Related NR4a1/Nur77 Suppression in Human Hepatic Stellate Cells In Vitro

**Figure 4:**
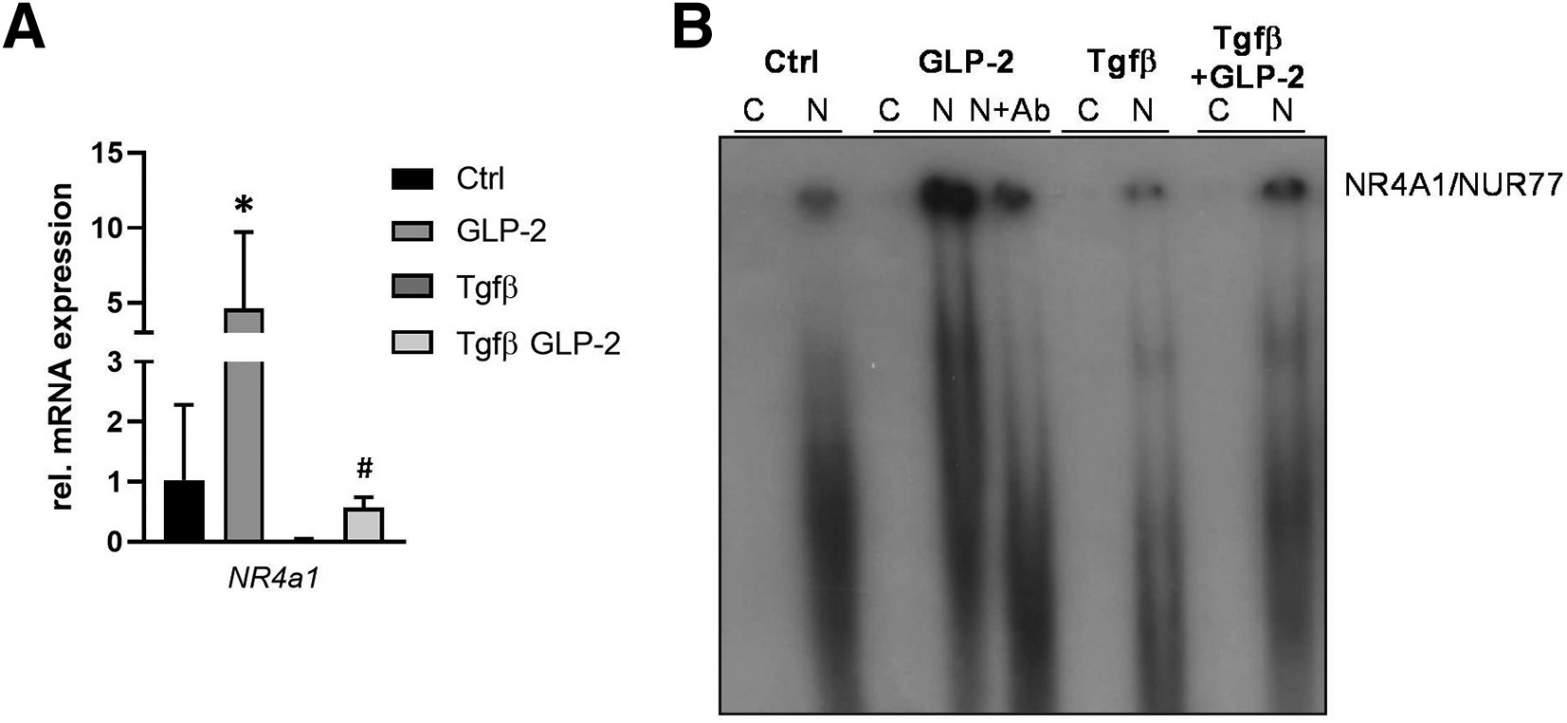
**GLP-2 treatment increases NR4a1/Nur77 expression and nuclear binding in human hepatic stellate cells in vitro.** (*A*) Real-time polymerase chain reaction was used to assess the mRNA expression of *NR4a1/Nur77* in the human hepatic stellate cell line LX-2. Tgfβ-induced suppression of *NR4a1/Nur77* could be counteracted by GLP-2 treatment. mRNA expression values were normalized against *36b4* levels and are shown relative to expression level in untreated control cells. ∗Significant difference from untreated control (Ctrl) cells. ^#^Significant difference from Tgfβ-treated cells. *P* < .05. (*B*) Representative electrophoretic mobility shift assay demonstrated that GLP-2 treatment increases the nuclear binding of NR4A1/NUR77, whereas Tgfβ challenge led to a reduction of the NR4A1/NUR77 nuclear binding. Of note, GLP-2 treatment was able to counteract the Tgfβ-related reduction of the NR4A1/NUR77 nuclear binding. Ab, antibody; C, cytoplasmatic protein fraction; N, nuclear protein fraction.

To investigate whether GLP-2 is also effective in counteracting HSC activation in humans, LX-2 cells were activated with Tgfβ (Figure 4*A*). As predicted, GLP-2 increased *NR4a1* mRNA expression, whereas Tgfβ treatment led to a significant reduction of *NR4a1* expression (Figure 4*A*). GLP-2 treatment was able to counteract the Tgfβ-related *NR4a1* suppression and restored normal levels of the transcription factor (Figure 4*A*). To assess the direct interaction of GLP-2 and NR4a1, an electrophoretic mobility shift assay was performed (Figure 4*B*). In contrast to Tgfβ, GLP-2 treatment was able to increase nuclear binding of NR4a1. Importantly, GLP-2 treatment was able to counteract Tgfβ-related NR4a1 suppression.

### GLP-2 Treatment Alters Hepatic Bile Acid Metabolism

**Figure 5:**
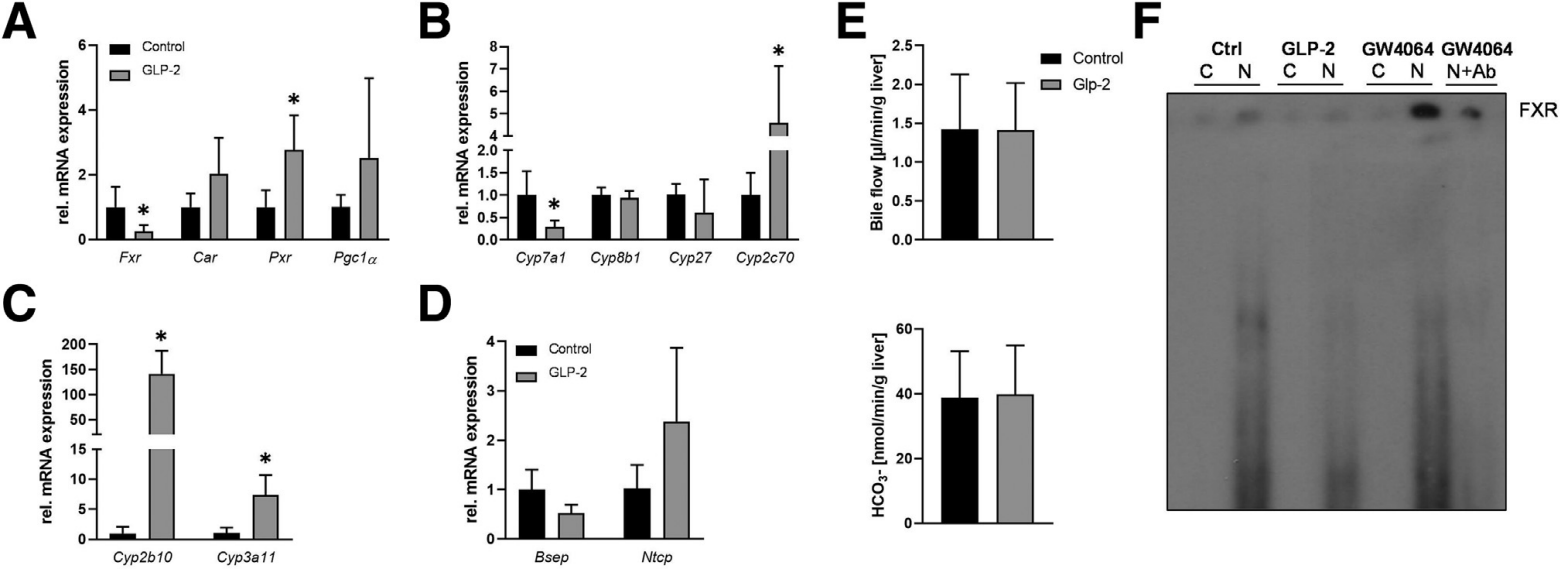
**GLP-2 treatment interferes with bile acid homeostasis in *Mdr2***^***-/-***^**mice.** Real-time polymerase chain reaction was used to assess the mRNA expression of (*A*) *Fxr*, *Car*, *Pxr*, and *Pgc1α*, (*B*) *Cyp7a1*, *Cyp8b1*, *Cyp27*, and *Cyp2c70*, (*C*) *Cyp2b10* and *Cyp3a11*, and (*D*) *Bsep* and *Ntcp* in the liver. mRNA levels of *Fxr* were significantly lowered, whereas *Car*, *Pxr*, and *Pgc1α* were increased. *Cyp7a1* was significantly lowered because of GLP-2 treatment in *Mdr2*^*-/-*^ mice, whereas *Cyp8b1* and *Cyp27* remained unchanged and *Cyp2c70* was increased. Gene expression of detoxification enzymes *Cyp2b10* and *Cyp3a11* was significantly increased because of GLP-2. Although *Bsep* expression levels tended to be lowered, *Ntcp* showed a tendential increase. mRNA expression values were normalized against *36b4* levels and are shown relative to the expression level in *Mdr2*^*-/-*^ control subjects. ∗Significant difference from *Mdr2*^*-/-*^ control mice; *P* < .05. (*E*) Hepatobiliary bile flow and bicarbonate (HCO_3_^-^) output were assessed. Neither bile flow nor HCO_3_^-^ output was changed because of GLP-2 treatment. (*F*) Representative electrophoretic mobility shift assay demonstrated that GLP-2 treatment suppresses the nuclear binding of FXR, whereas GW4064 (FXR agonist) challenge led to an increase of FXR nuclear binding. Ab, antibody; C, cytoplasmatic protein fraction; N, nuclear protein fraction.

Next, we explored whether GLP-2 also impacts on BA homeostasis and signaling in addition to its antifibrotic function in HSCs. Despite reduced hepatic *Fxr* expression (Figure 5*A*), *Cyp7a1* mRNA levels were significantly repressed by GLP-2. *Cyp8b1* and *Cyp27* remained unchanged but *Cyp2c70* was increased upon GLP-2 challenge (Figure 5*B*). In line with this, increased expression of *Cyp2c70* increased the abundance of muricholic acids in *Mdr2*^*-/-*^ mice subjected to GLP-2 treatment (Table 1). Expression of the nuclear receptor *Pxr* was markedly increased in the presence of GLP-2, whereas expression of *Car* and its coactivator *Pgc1*α was unchanged (Figure 5*A*). The gene expression of the constitutive androstane receptor and pregnane-X-receptor downstream targets *Cyp2b10* and *Cyp3a11* was increased in *Mdr2*^*-/-*^ mice treated with GLP-2 (Figure 5*C*). Farnesoid X receptor (FXR) targets, such as *Bsep* and *Ntcp*, tended to be reduced and increased, respectively (Figure 5*D*). However, hepatobiliary bile flow and biliary HCO_3_^-^ output remained unchanged among the groups (Figure 5*E*).

**Table 1:** Total and Individual Intrahepatic Bile Acid Levels

pmol/mg liver	Mdr2^-/-^ Ctrl	Mdr2^-/-^ GLP-2
ToMCA	23.9 ± 4.2	81.93 ± 52.2
TαMCA	5.5 ± 1.4	4.2 ± 2.4
TβMCA	67.2 ± 21.3	158.4 ± 21.3^a^
TCA	106.0 ± 15.3	211.3 ± 108.9
TUDCA	1.0 ± 0.2	1.1 ± 0.6
TCDCA	5.5 ± 0.59	8.5 ± 3.2
TDCA	1.7 ± 0.4	1.4 ± 1.0
oMCA	5.4 ± 2.2	16.4 ± 4.76^a^
αMCA	1.3 ± 0.9	1.8 ± 1.0
βMCA	19.3 ± 5.2	47.5 ± 12.0^a^
Total	240.1 ± 27.8	527.5 ± 312.6

αMCA, alpha muricholic acid; βMCA, beta muricholic acid; oMCA, omega muricholic acid; TCA, taurocholic acid; TCDCA, taurochenodeoxycholic acid; TDCA, taurodeoxycholic acid; TαMCA, tauro-alpha muricholic acid; TβMCA, tauro-beta muricholic acid; ToMCA, tauro-omega muricholic acid; TUDCA, tauroursodeoxycholic acid.

a Significantly different from Mdr2^-/-^ Ctrl. *P* ≤ .05 was considered significant.

### GLP-2 Treatment Suppresses Hepatic FXR Nuclear Binding in Human Hepatocytes In Vitro

To examine the mechanisms of reduced *Fxr* mRNA expression observed in GLP-2-treated *Mdr2*^*-/-*^ mice and whether reduced expression results in reduced FXR function and subsequent signaling, electrophoretic mobility shift assay was performed in immortalized human hepatocytes cells treated with GLP-2 and GW4064 (FXR agonist, positive control) (Figure 5*F*). GW4064 treatment increased the nuclear binding of FXR, whereas GLP-2 led to a reduction of FXR nuclear binding (Figure 5*F*).

### GLP-2 Treatment Increases Intestinal *Fgf15* Expression

**Figure 6:**
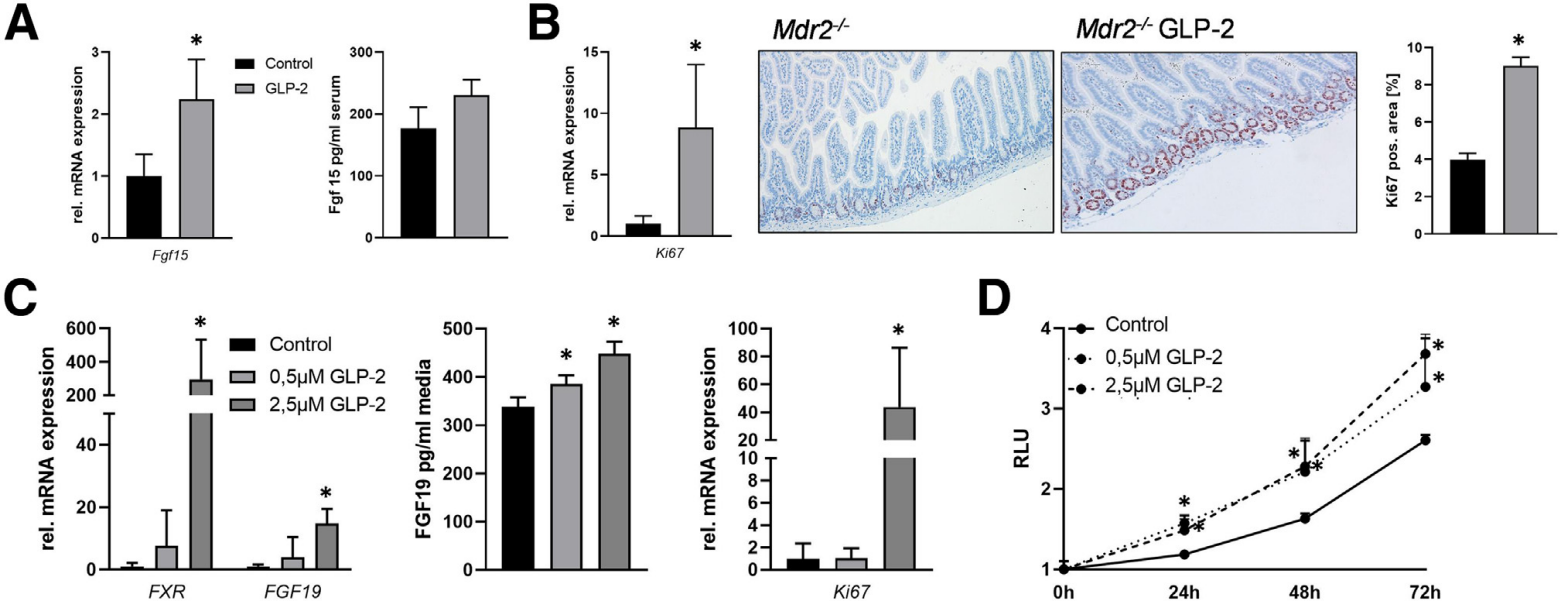
**GLP-2 treatment activates intestinal FGF15/19 expression.** Real-time polymerase chain reaction was used to assess the intestinal mRNA expression of (*A*) *Fgf15* and (B) *Ki67*. Fgf15 enzyme-linked immunosorbent assay (ELISA) was used to investigate Fgf15 levels in systemic blood of *Mdr2*^*-/-*^ mice on GLP-2 treatment (*A*). Intestinal *Fgf15* mRNA and protein expression as well as *Ki67* mRNA levels were elevated in *Mdr2*^*-/-*^ mice on GLP-2 treatment. Accordingly, Ki67 immunohistochemistry (×10 magnification) (*B*) showed an increase in Ki67-positive cells numbers in the intestine of *Mdr2*^*-/-*^ mice treated with GLP-2. mRNA expression values were normalized against *18sRNA* levels and are shown relative to the expression level in *Mdr2*^*-/-*^ control animals. ∗Significant difference from *Mdr2*^*-/-*^ control mice; *P* < .05. (*C*) Real-time polymerase chain reaction was used to assess the mRNA expression of *FXR*, *FGF19*, and *KI67* in human-derived intestinal organoids treated with GLP-2. FGF19 ELISA was used to assess FGF19 protein concentration in cell culture supernatant. Gene expression of the aforementioned genes was significantly increased by GLP-2 treatment. mRNA expression values were normalized against *18sRNA* levels and are shown relative to expression level in untreated control subjects. FGF19 levels were significantly increased in supernatant of organoids treated with 0.5 μM and 2.5 μM GLP-2. ∗Significant difference from untreated control cells; *P* < .05. (*D*) CellTiter-Glo Luminescent Cell Viability Assay was performed to assess cell proliferation in human-derived intestinal organoids. GLP-2 significantly increased cell proliferation independent of the used concentrations. ∗Significant difference from untreated control cells; *P* < .05.

Because in *Mdr2*^*-/-*^ mice treated with GLP-2 *Fxr* and *Cyp7a1* mRNA expression was reduced, we next explored intestinal *Fgf15* gene expression. In line with low hepatic *Cyp7a1* levels, intestinal *Fgf15* expression was increased on GLP-2 treatment (Figure 6*A*). Accordingly, serum levels of Fgf15 were tendentially increased in *Mdr2*^*-/-*^ mice treated with GLP-2 (Figure 6*A*). In consistence with GLP-2 effects known from literature, the proliferation marker *Ki67* was increased at mRNA and protein level (Figure 6*B*), suggesting that increased proliferation might be linked to increased *Fgf15* secretion.

### GLP-2 Treatment Increases *FXR-FGF19* Signaling Axis in Human Intestinal Organoids

To verify whether our findings in murine intestines translate to humans, human-derived intestinal organoids (expressing GLP-2r at a Ct value of 33.4 in average) were treated with GLP-2 over 24 hours. Gene expression profiling revealed a significant increase in *FXR* and *FGF19* mRNA expression and fibroblast growth factor (FGF)19 levels in cell culture supernatant because of GLP-2 treatment (Figure 6*C*), indicating a direct effect of GLP-2 on the FXR-FGF19 signaling axis. In addition, GLP-2 treatment increases also proliferation of human intestinal organoids shown by increased Ki67 mRNA levels and by the proliferation assay (Figure 6*C* and *D*).

## Discussion

In this study we demonstrated that the GLP-2 analogue teduglutide improves hepatic inflammation and fibrosis in the *Mdr2*^*-/-*^ mouse model of sclerosing cholangitis. Our observations demonstrate that GLP-2 treatment may be a novel therapeutic approach to counteract hepatic inflammation and fibrosis in the context of cholestasis beyond its role as proproliferative agent serving primarily in the gut. Herein we provide evidence that GLP-2 receptor signaling found to be present in HSCs^6^^,^^7^ improves liver injury via increasing NR4a1 nuclear binding and subsequent antifibrotic function in HSCs. Moreover, GLP-2 exerts Cyp7a1 inhibitory effects via activating intestinal FXR-FGF15/19 signaling axis. Presence of GLP-2 in the system leads also to increased expression of Cyp2c70, thus promoting muricholic acid synthesis, thereby restoring a less toxic BA homeostasis.

NR4a1 was identified as a key target to control fibrogenesis^10^ and loss of GLP-2 signaling results in activation of HSCs.^6^ Our findings in whole liver and in primary HSCs isolated from *Mdr2*^*-/-*^ mice subjected to GLP-2 treatment discovered antifibrotic function of the GLP-2 analogue teduglutide caused by activation of NR4a1 expression. Furthermore, electrophoretic mobility shift assay performed in the human HSC cell line LX-2, activated with Tgfβ revealed that GLP-2 treatment was able to restore Tgfβ-related reduction of NR4a1 nuclear binding further underlining the antifibrotic function of teduglutide in HSCs.

In hepatocytes, presence of GLP-2 led to changes in hepatic BA homeostasis resulting in a less toxic bile pool mainly consisting of muricholic acids. In line with increased muricholic acid levels, known to serve as FXR antagonist,^16^ hepatic FXR expression found in GLP-2-treated animals was reduced. In addition to the potential muricholic acid–related reduction we observed that treatment with GLP-2 reduced FXR nuclear binding in vitro. This finding may be of particular relevance to translate our findings into the human situation because levels of muricholic acids in human are neglectable. Of note, also the discrepancy that FXR expression is increased in piglets subjected to GLP-2^17^ could be attributed to the fact that muricholic acids are mainly present in mice. Despite reduced FXR activity, Cyp7a1 (normally repressed by FXR) was also reduced because of GLP-2 treatment. This phenomenon might be explained by increased intestinal Fgf15/19 expression caused by GLP-2 treatment. Accordingly, *FXR* and *FGF15/19* mRNA expression and FGF15/19 levels in mouse serum and cell culture supernatant were increased in GLP-2-treated *Mdr2*^*-/-*^ mice and human-derived intestinal organoids, respectively. These finding argue for a direct GLP-2-related activation of the FXR-FGF19 signaling axis. Furthermore, increased proliferation was also observed, possibly also contributing to increased FGF19 expression/secretion because of increased mass of enterocytes based on GLP-2 treatment.^18^

Reduced intrahepatic FXR signaling could also explain the trend toward elevated intrahepatic total BA levels because it regulates hepatic BA uptake (Na^+^-taurocholate cotransporting polypeptide) and export (bile salt export pump). Reduced expression of Bsep, the main gatekeeper for hepatic BA export, could also be relevant for the finding of unchanged bile flow, which is in line with what has been shown previously.^19^ Moreover, reduced gallbladder contraction caused by the presence of GLP-2 (as reported previously^19^) may also be relevant for unchanged levels of hepatobiliary bile flow and increased levels of intrahepatic BAs.

Decreased hepatic FXR signaling in the context of cholestatic liver disease might be seen as critical because FXR agonists are currently exported as therapeutic strategies in PSC^20^ and have been shown to be an effective second-line treatment in primary biliary cholangitis.^20^ However, suppression of *Cyp7a1* as main therapeutic target of FXR was also achieved by GLP-2 via activation of the FXR–FGF15/19 signaling axis. Thus, a combination of FXR agonists, such as obeticholic acid or cilofexor, and antifibrotic drugs, such as GLP-2 analogues, could be considered as future perspective in counteracting cholestatic liver diseases. Furthermore, the combination of FXR agonists and GLP-2 analogues may also be attractive for patients suffering from PSC accompanied by inflammatory bowel disease, because it has been shown that GLP-2 treatment has anti-inflammatory effects in a mouse model of inflammatory bowel disease.^21^

Because GLP-2 receptor expression was not found in murine cholangiocytes,^22^ the observation of reduced cholangiocyte activation might be an indirect effect of GLP-2 treatment. Activated HSCs secrete proinflammatory cytokines and chemokines,^11^ which were shown to contribute to activation of cholangiocytes,^12^ thus the GLP-2-related deceleration of HSC activation may, in addition to the beneficial changes in the BA profile, contribute to the reduced reactive cholangiocyte phenotype observed in *Mdr2*^*-/-*^ mice subjected to GLP-2 treatment.

In conclusion, we demonstrated that activation of NR4a1 via teduglutide improves hepatic inflammation and fibrosis in the *Mdr2*^*-/-*^ mouse model of sclerosing cholangitis. Additionally, teduglutide treatment modified the intrahepatic BA composition toward a more favorable hydrophilic direction, which at least in part, could also contribute to improvement of liver and bile duct injury and fibrosis.

## Materials and Methods

### Animals

*Eight-week-old male FVB/N Mdr2/Abcb4−/−* mice obtained from Jackson Laboratory (Bar Habor, ME) were injected intraperitoneally with the Glp-2 analogue teduglutide 0.05 mg/kg daily for 4 weeks. Animals were housed in a 12-hour light/dark house facility with water and standard chow diet (SSNIFF, Soest, Germany) *ad libitum*. The experimental procedures were approved by the Animal Ethics Committee of the Medical University of Vienna and the Federal Ministry of Science, Research and Economy (BMWFW-66.009/0315-WF/V/3b/2014) and performed according to the Animal Research: Reporting of In Vivo Experiments (ARRIVE) guidelines.

### Hepatic Stellate Cell Isolation

HSCs were isolated from mouse livers as described before for rats with small modifications, adapting the protocol to mice.^23^ Briefly, livers of anesthetized mice were perfused first with 20 mL preperfusion buffer (HBSS, Thermo Fisher Scientific, Waltham, MA; + 1% heparin, Gilvasan Pharma), followed by 40 mL perfusion buffer A (HBSS + 0.001% DNAse + 0.015% collagenase A + 0.15% pronase, all Merck, Darmstadt, Germany). Digested livers were excised, minced, and further digested in 20 mL buffer B (HBSS + 0.0005% DNAse + 0.01% collagenase A + 0.04% pronase) in vitro at 37°C for 5 minutes. For cirrhotic livers, 30 % extra collagenase A was added to both buffers. The suspension was passed through a 100 μm cell strainer into 20 mL ice cold HBSS. After centrifugation, the nonparenchymal cells of 3 mice were pooled and subjected to a density gradient centrifugation using 11.5% OptiPrep (Merck) to purify HSCs, which were plated overnight into 6-well plates, before lysing them in TriFast (VWR, Radnor, PA) for RNA extraction.

### Routine Serum Biochemistry and Histology

Serum biochemistry and histologic stainings (hematoxylin-eosin, Sirius red) were performed as described previously.^24^

### Immunohistochemistry

Detection of hepatic MAC-2 and osteopontin was performed as described previously.^25^^,^^26^

### Hepatic Bile Acid Analysis

Hepatic BA profiles were acquired using ultraperformance liquid chromatography tandem mass spectrometry as described previously.^27^^,^^28^

### Messenger RNA Analysis and Polymerase Chain Reaction

RNA isolation from liver and primary HSCs, complementary DNA synthesis, and real-time polymerase chain reactions were performed as described previously.^29^ Oligonucleotide sequences are available on request.

### Measurement of Bile Flow

Bile flow and hepatobiliary bicarbonate (HCO_3_^-^) measurements were performed as described previously.^1^ In brief, the common bile duct was ligated and the gallbladder was cannulated. After an equilibration period, bile was collected in preweighted tubes for 20 minutes. Bile flow was determined gravimetrically and normalized to liver weight. Biliary bicarbonate concentrations were measured in a routine laboratory and normalized to bile flow.

### Cell Culture

LX-2 cells, an immortalized HSC line,^30^ kindly provided by Professor S.L. Friedman (Mount Sinai School of Medicine, New York, NY) were cultured with Dulbecco's modified Eagle medium (Life Technologies) supplemented with 5% non-heat-inactivated fetal bovine serum and 1% penicillin/streptomycin solution (EuroClone). Cells were treated with 2.5μM GLP-2 and Tgfβ for 36 hours. Immortalized human hepatocytes^31^ were cultured with Dulbecco's modified Eagle medium supplemented with 10% heat-inactivated fetal bovine serum and 1% penicillin/streptomycin solution (EuroClone). Cells were treated with 2.5 μM Glp-2 and GW4064 for o36 hours.

### Electrophoretic Mobility Shift Assay

Cytosolic and nuclear extracts from LX-2 cells treated with 2.5 μM GLP-2 and Tgfβ and from immortalized human hepatocytes treated with 2.5 μM GLP-2 and GW5064 were obtained after lysis with 1% Nonidet P40 and specific buffer (25 mM 4-[2-hydroxyethyl]-1-piperazine ethanesulfonic acid [pH 7.9], 50 mM NaCl, 5% glycerol, and 0.5 mM dithiothreitol) containing protease inhibitors. Double-stranded NR4a1 response element were labeled with P^32^adenosine triphosphate, purified using the QIAquick Nucleotide Removal kit (Qiagen, Germany), and incubated with a binding buffer (10 mM tris-hydroxymethyl aminomethane [pH 8.0], 40 mM KCl, 0.05% Nonidet P40, 6% glycerol). Four micrograms of either cytosolic or nuclear extract were incubated with labeled probes and buffer for 10 minutes at room temperature and loaded on 4% acrylamide/bis-acrylamide gel. After the run, gel was dried for 1 hour at 60°C and transferred into a developing cassette (Biomax, Kodak) for overnight film exposure at –80°C.

### Organoid Isolation from Human Biopsies and Organoid Cultures

Ileal biopsies from unaffected areas of the small intestine were taken in the operating room during small intestinal resections of patients with Crohn’s disease after informed consent and institutional review board approval (EK# 1915/2021) at the Division of Visceral Surgery, Department of General Surgery, Medical University of Vienna. On sample collection, they were immediately transported to the laboratory for processing. The biopsy was cut into pieces and incubated by rotating in chelating solution at 4°C for 30 minutes. Afterwards, glands were squeezed out of the tissues pieces by applying pressure on a glass slide placed on top. Glands were then collected for cell digestion 5 minutes in trypsin.^32^^,^^33^ Intestinal crypts were resuspended in Cultrex Basement Membrane Extract (R&D Systems) and seeded in 40–50 μL droplets into multiwell plates. Organoid culture medium containing growth factors and supplements was gently added onto Basement Membrane Extract drops and incubated at 37°C, 5% CO_2_, 95% air, and 95 % humidity.^32^^,^^34^ For the first 3 days of establishing new cultures, fungin (InvivoGen) was added to prevent fungal contamination during culture initiation and RhoK inhibitor is added to increase survival of cells and to promote expansion of stem cells in culture.^32^ Ileal organoids were passaged 1 time a week using a 0.05 % trypsin/EDTA solution^33^ (TrypLE Express, Gibco). During every passage, RhoK inhibitor was added but not required when medium was exchanged.^32^ The human intestinal organoids were treated with 0.5 μM and 2.5 μM GLP-2 for 24 hours. RNA was isolated using the Sigma-Aldrich RNA isolation kit (RTN350-1KT) according to the manufacturer’s protocol. Another batch of organoids treated with Glp-2 was used for the CellTiter-Glo Luminescent Cell Viability Assay (Promega, #G7570). The assay was performed according to the manufacturer’s protocol.

### FGF15/19 Enzyme-Linked Immunosorbent Assay from Mouse Serum and Cell Culture Supernatant

FGF15 enzyme-linked immunosorbent assay was performed from serum obtained from venous blood of the animals. Enzyme-linked immunosorbent assay was performed according to manufacturer’s protocol (FineTest, EM0286). FGF19 enzyme-linked immunosorbent assay was performed from cell culture supernatant taken after 24-hour treatment of human intestinal organoids with 0.5 μM and 2.5 μM GLP-2. FGF19 enzyme-linked immunosorbent assay from Sigma-Aldrich (RAB0540-1KT) was performed according to the manufacturer’s protocol.

### Statistical Analysis

Results were evaluated using GraphPad Prism 8.4.1. Statistical analysis was performed using 2-way analysis of variance. Data were reported as means of 5–7 animals per group ± standard deviation. In vitro experiments were performed 2 times with 3 biologic replicates (and 2 technical replicates). A *P* ≤ .05 was considered significant.
